# Acute-Phase Stroke Outcome and Lipids

**DOI:** 10.14744/SEMB.2020.26817

**Published:** 2021-12-29

**Authors:** Osman Serhat Tokgoz, Figen Guney, Ahmet Kaya, Ahmet Bugrul, Esra Eruyar, Huseyin Buyukgol, Abdullah Seyithanoglu, Mehmet Sinan Iyisoy

**Affiliations:** 1. Department of Neurology, Necmettin Erbakan University, Meram Medical Faculty, Konya, Turkey; 2. Department of Endocrinology, Baskent University Hospital, Konya, Turkey; 3. Department of Neurology, Lokman Hekim Akay Hospital, Ankara, Turkey; 4. Department of Neurology, KTO Karatay University, Medical Faculty, Konya Medicana Hospital, Konya, Turkey; 5. Department of Neurology, Kahramanmaras, Necip Fazıl City Hospital, Kahramanmaras, Turkey; 6. Department of Medical Education and Informatics, Necmettin Erbakan University, Meram Medical Faculty, Konya, Turkey

**Keywords:** Malnutrition, mortality, stroke, triglyceride

## Abstract

**Objectives:**

The aim of the study is to investigate the relationship of lipid subgroups with short-term mortality in acute stroke (AS).

**Methods:**

This retrospective study included 698 patients with AS who presented within 24 h of symptom onset. A hemogram from peripheral venous blood samples was taken at admission. Total cholesterol (TC), triglyceride (TG), high-density lipoprotein cholesterol (HDL-C), low-density lipoprotein cholesterol (LDL-C), TC/HDL-C rate, and TG/HDL-C rate were recorded. Duration of follow-up was defined as 30 days.

**Results:**

64 out of 698 patients died during the follow-up period. The mean TG, TG/HDL-C, and TC/HDL-C levels were significantly lower in the mortality group than the survival group. In the receiver operating characteristic (ROC) analysis, the cutoff values and area under the curve of the TG, TG/HDL-C, TC, and TC/HDL-C levels for short-term stroke mortality are as follows ([100.2 mg/dL, 0.648]; [2.52, 0.650]; [170.50 mg/dL, 0.598]; and [4.32, 0.640], respectively). In the Cox regression model, only TG and TG/HDL-C, according to their ROC cutoff values, were independent variables as short-term mortality predictors (TG ≤100.2 mg/dL, HR:2.413 , 95% CI: 1.345–4.327, P:0.004); (TG/HDL ≤2.56, HR: 2.720, 95% CI: 1.389–5.359, P:0.003, respectively).

**Conclusion:**

Dyslipidemia is a well-known as a risk factor of stroke. However, this study focused on the estimation that lower TG and TG/HDL-C levels at the time of hospital admission might be predictors of short-term mortality within a month of AS attack, which is a different subject from long term risk factors of stroke. Serum TG level may be a better indicator for mortality in the acute hypercatabolic trauma such as stroke.

Stroke is a worldwide disease causing mortality and morbidity. So many studies are conducted to find predictor factors of long-term outcome after acute stroke (AS) in literature.^[1]^ Stroke severity, age, cardiac disease, cardio-embolic etiology, and hypertension and diabetes mellitus are the most important prognostic factors of long-term outcomes in AS. The identification of the other modifiable prognostic factors allows selection of appropriate treatment.^[2]^

Dyslipidemia is another definite etiologic factor for vascular disease. Hyperlipidemia is a well-documented risk factor for cardiovascular disease.^[3]^ Cholesterol and triglyceride (TG) are an integral of risk prediction and prevention models for cardiovascular disease.^[4-7]^ But the association between cholesterol and ischemic stroke is controversial. Some studies have suggested that high cholesterol is associated with a higher risk for ischemic and hemorrhagic stroke (HS)^[8,9]^ while many studies have shown that a low cholesterol levels were associated with higher stroke mortality.^[3,10-12]^ On the other hand, the prospective cohort studies consistently showed inverse associations of cholesterol with HS, especially intracerebral hemorrhage (ICH).^[13,14]^

The associations between AS outcome and lipid subgroups such as total cholesterol (TC), low-density lipoprotein cholesterol (LDL-C), high-density lipoprotein cholesterol (HDL-C), TG, TG/HDL-C, and TC/HDL-C ratios have generated controversy in literature.^[2]^ We aimed to examine the effects of these parameters, which are well-known stroke risk factors, on AS short-term outcomes in the Anatolian population. Acute term stroke is an acute metabolic stress period and effects of lipid subgroups on mortality in acute stress period will be examined, unlike classical risk factor studies which study the relation between the parameters and stroke risk.

## Methods

### Study Population

The study is a hospital-based, retrospective, follow-up study. 1024 patients with AS (>18 years of age) were screened between January 2004 and January 2015. Patients within the 1^st^ day of admission were included in the study. The study protocol was approved by the local ethics committee (2011/185) and based on the Declaration of Helsinki.

**Figure 1.**
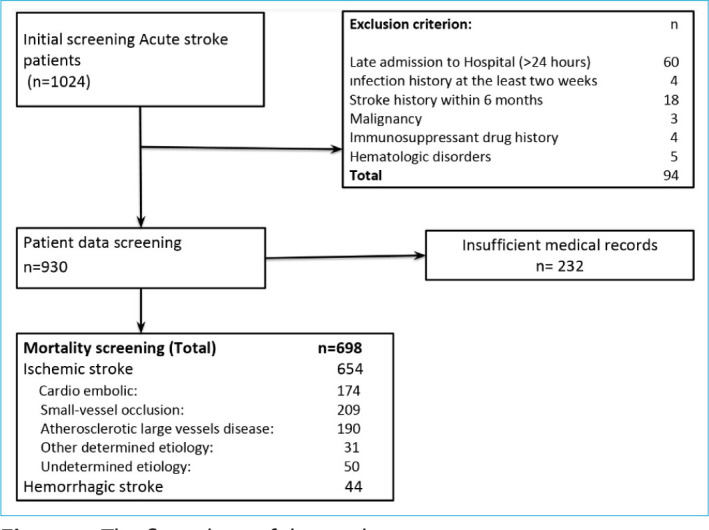
The flow chart of the study.

The flow chart of the exclusion criteria is shown in Figure 1. The patients who were admitted to the hospital >24 h after AS, an infection history within 2 weeks, malignancy, stroke history within 6 months, immunosuppressant drug history, and hematologic disorders were excluded from the study.

### Study Protocol

All cases were screened using the International Classification of diseases, 10^th^ revision code “I 61.0–2” from the database of the hospital’s electronic record system. 698 patients met the criteria. The demographic and clinical characteristics of the patients were obtained from the patient’s archived records at admission. Stroke outcome was evaluated at 1 month after AS according to the modified Rankin Scale and Glasgow Coma Score. Hospital mortalities of patients were determined according to the medical record. Patients who were discharged from hospital before 30 days were followed up through the outpatient department visit records (Fig. 1).

### Statistical Analysis

Data were analyzed using SPSS software (version 15.0; SPSS Inc, Chicago, IL) and presented as mean±standard deviation (sd) or median (95% confidence interval, CI). Distribution normality was analyzed with the Kolmogorov–Smirnov test. The Student t-test for normally distributed variables and the Mann–Whitney U-test for the non-parametrically distributed variables were used. The difference between categorical variables was determined by the χ^2^ test. Mixed models were used to compare the albumin 1 and 2 scores of the mortality and surviving groups. The Cochrane Armitage test was used for the comparison the mortality rates in both the TC (≤100; 101–150; 151–200; >200 mg/dL) and TG (≤100; 101–200; >200 mg/dL), respectively. Receiver operating characteristic (ROC) curves were used to evaluate the distinguished capacities of the areas under the curve (AUCs) and 95% CIs for TC, TC-HDL, TG, TG/HDL, and decrease in albumin blood level. Kaplan–Meier survival analysis for TG-HDL rate, TC-HDL rate, TG, and TC was estimated according to their ROC cutoff values with log rank test. A Cox regression model for acute-term stroke outcomes was used to evaluate Hazard ratios (HRs) and corresponding 95% CIs according to their ROC cutoff values. P<0.05 was considered as statistically significant.

## Results

The mean ages of the surviving and dead patients with AS were 65.96±13.61 and 73.06±11.11, respectively.

The mortality rate was 9.16 % (n=64) by 30 days of follow-up. Demographic and laboratory findings of the surviving and dead patients are reported in Table 1. Our patients had taken about 25–30 kcal/kg in the following period in the study. The TG, TG/HDL-C, and TC/HDL-C on admission to the hospital of patients who died were significantly lower than in the surviving group, while white blood cell and C reactive protein were higher (Table 1). There were no significant differences between ischemic and HS regarding TC, TG, HDL, and LDL values (z= −0.011, p=0.991; z= −0.002, p=0.999; z= −0.406, p=0.684; and z= −0.260, p=0.495, respectively).

**Table 1.** Demographic and laboratory findings of the patients

	**Surviving (n=634)**	**Dead (n=64)**	**p**	**χ^2^**
Age, (mean sd, p, z value)	65.96 (13.61)	73.06 (11.11)	<0.0001	−4.202
Sex (Female, %)	291 (41.7)	34 (53.1)	0.483	1.455
Diabetes mellitus (%)	189 (29.9)	20 (31.2)	0.886	0.050
Stroke history (%)	192 (30.3)	43 (67.2)	<0.0001	35.450
Hypertension (%)	390 (61.7)	50 (78.1)	0.001	6.735
CAD (%)	89 (14.0)	23 (35.0)	<0.0001	20.696
Stroke type (infarct%)	604 (95.3)	50 (78.1)	<0.0001	28.925
Drugs				
Antihypertensive	461 (58.8)	69 (75.8)	0.003	10.69
ASA	31 (4.9)	5 (7.9)	0.363	1.065
Clopidogrel	20 (3.2)	2 (3.1)	1.000	0.000
Coumadin	37 (5.8)	7 (10.9)	0.110	2.562
Oral antidiabetic	68 (10.7)	4 (6.2)	0.386	1.259
Insulin	34 (5.4)	4 (6.2)	0.771	0.087
Statin	28 (3.6)	0	0.099	2.954
AF	60 (9.6)	0	0.004	6.707
	Z			
WBC (10^3^/mL), (median, 25–75%)	8.6 (7.0–10.8)	11.00 (8.8–14.4)	<0.0001	−4.9
Hg (median, 25–75%)	13.20 (11.9–14.4)	13.1 (11.9–14.5)	0.816	−0.23
Platelet (median, 25–75%)	243.00 (198.5–295.5)	245.00 (176.0–289.6)	0.360	−0.915
CRP (median, 25–75%)	20.0 (10.0–33.8)	38.20 (17.9–85.4)	<0.0001	−5.25
Sedimentation (median, 25–75%)	28.00 (21.0–43.0)	23.00 (16.0–50.0)	0.064	−1.85
Glucose (mg/dL), (median, 25–75%)	113.00 (94.0153.8)	141.00 (114.3–190.0)	<0.0001	−4.08
Creatinine (mg/dL) (median, 25–75%)	0.9 (0.7–1.1)	1.00 (0.8–1.3)	0.016	−2.42
TC (mg/dL) (median, 25–75%)	177.50 (145.0–203.3)	168.50 (144.3–195.0)	0.163	−1.40
TG (mg/dL) (median, 25–75%)	119.00 (81.8–167.3)	90.00 (66.7–121.3)	<0.0001	−3.85
LDL (mg/dL), (median, 25–75%)	112.2 (87.2–133.5)	112.1 (86.4–131.9)	0.452	−0.75
HDL (mg/dL) (median, 25–75%)	36.00 (29.2–44)	39.0 (29.3–48.0)	0.070	−1.81
TC/HDL (median, 25–75%)	4.82 (3.9–6.0)	4.25 (3.6–5.2)	0.004	−2.91
TG/HDL (median, 25–75%)	3.27 (2.0–5.0)	2.17 (1.4–4.0)	<0.0001	−3.72
ALT (median, 25–75%)	19.00 (15–25.0)	20.00 (14.0–28.0)	0.517	−0.65
Albumin (g/L, 25–75%)	3.7 (3.4–4)	3.7 (3.3–4.0)	0.495	−0.682
Glasgow coma score (median, 25–75%)	15.00 (15.0–15.0)	11.00 (6.8–13.0)	<0.0001	−13.10
Modified Rankin score (median, 25–75%)	2.00 (1.0–4.0)	5.00 (4.0–5.0)	<0.0001	−8.19

CAD: Coronary artery disease; ASA: Acetylsalicylic acid; AF: Atrial fibrillation; WBC: White blood cell; Hg: Hemoglobin; CRP: C reactive protein; TC: Total cholesterol; TG: Triglyceride; LDL: Low density lipoprotein; HDL: High density lipoprotein.

The mortality rates are shown in Table 2 according to tertiles for TG and TC. Mortality rates at low TG and TC tertiles were significantly higher than high TG and TC tertiles (Z: 4.61, χ^2^: 22.24, p<0.001) and the TC tertiles (Z: 4.23, χ^2^: 18.34, p<0.001) (Table 2).

**Table 2.** Mortality rate according to the tertiles (Cochran Armatage test)

	**mg/dL**	**Mortality rate (%)**	**Z/χ^2^**	**P**
TG	≤100	14.78	4.61/22.24	<0.0001*
	101–200	6.38		
	>201	2.63		
TC	≤100	17.24	4.23/18.34	<0.0001*
	101–150	11.28		
	151–200	8.67		
	>201	4.42		

*Lower tertiles of both TG and TC are significantly associated with higher mortality. TC: Total cholesterol; TG: Triglyceride.

**Figure 2.**
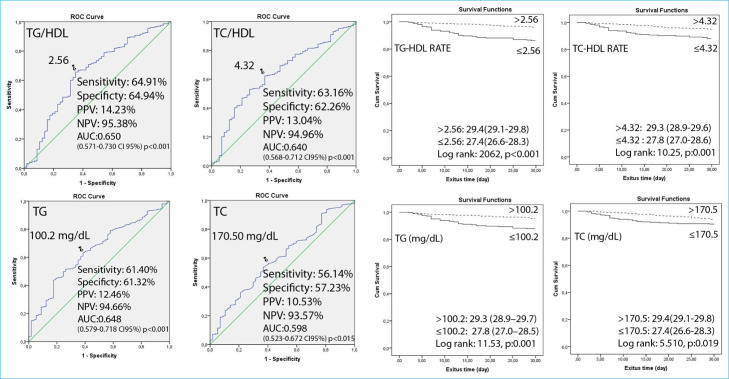
**(a)** ROC analysis of TG-HDL rate, TC-HDL rate, TG, and TC for short-term mortality. **(b)** Kaplan–Meier survival estimates for TG-HDL rate, TC-HDL rate, TG, and TC in acute stroke and a comparison of survival for these parameters according to the their ROC cutoff values with log rank test. PPV: Positive predictive value; NPV: Negative predictive value; AUC: Area under curve; CI: Confidence interval; TG/HDL rate: Triglyceride/high density lipoprotein rate; TC/HDL: Total cholesterol/high density lipoprotein rate; TG: Triglyceride; TC: Total cholesterol.

These ROC cutoff values of TG, TG/HDL-C, TC, and TC/HDL-C for short-term mortality were 100.2 mg/dL; 2.56; 170.5 mg/dL; 4.32, respectively (Fig. 2A). The AUC values were 0.648 for TG, 0.650 for TG/HDL-C, 0.640 for TC/HDL-C, and 0.598 for TC (Fig. 2A).

When analysis of 30-day survival period of patients was examined according to the ROC cutoff values of TC, TG, TG/HDL-C, and TC/HDL-C, the Kaplan–Meier survival curves were different between the two groups (Fig. 2B).

TG (≤100.2 mg/dL,), TG/HDL-C (≤2.56), coronary artery disease, age, and glucose were independent predictors of mortality in the Cox regression analysis (Table 3). The HRs for TG and TG/HDL-C values were also an independent variable as short-term mortality predictors (TG ≤100.2 mg/dL, HR: 2.413, 95% CI: 1.345–4.327, p=0.004); (TG/HDL ≤2.56, HR: 2.720, 95% CI: 1.389–5.359, p=0.003, respectively). TC and TC/HDL values were not independent predictors (TC ≤170.5 mg/dL, HR:1.484, 95% CI: 0.826–2.665, p=0.186); (TC/HDL ≤4.32, HR: 1.202, 95%CI: 0,619–2.333, p=0.586) (Table 3).

**Table 3.** Cox regression results for the predictors of mortality

	**Model 1**		**Model 2**	
	**HR (95,0% CI)**	**P**	**HR (95,0% CI)**	**P**
Age	1.031 (1.003–1.059)	0.028*	1.032 (1.004–1.061)	0.024*
Hypertension	1.655 (0.853–3.211)	0.136	1.694 (0.872–3.289)	0.120
CAD	3.024 (1.719–5.319)	<0.0001*	3.075 (1.740–5.434)	<0.0001*
Glucose	1.004 (1.001–1.007)	0.009*	1.004 (1.001–1008)	0.009*
TG/HDL (≤2.56)	2.720 (1.389–5.359)	0.004*		
TC/HDL (≤4.32)	1.202 (0.619–2.333)	0.586		
TG (≤100.2 mg/dL)			2.413 (1.345–4.327)	0.003*
TC (≤170.5 mg/dL)			1.484 (0.826–2.665)	0.186

HR: Hazard ration; CI: Confidential interval; CAD: Coronary artery disease; TC: Total cholesterol; TG: Triglyceride; TC/HDL: Total cholesterol/high density lipoprotein rate; TG/HDL rate: Triglyceride/high density lipoprotein rate. *Independent prognostic factor on acute-term stroke mortality. The TG and TC values were added to model after TC/HDL, and TG/HDL values extracted, which are similar structure (model 2).

## Discussion

The present study has shown that the TG, TG/HDL-C, and TC/HDL-C levels are significantly lower in the mortality group than the group of surviving patients. However, only the low TG, TG/HDL-C levels are independent predictors for mortality. There is not any significant association between mortality and the other lipid parameters.

Stroke is the third leading cause of death in the world, and high plasma lipid concentrations are one of the definitive risk factors for atherosclerosis. The accumulation of lipids within the arterial wall has been accused for atherosclerosis pathogenesis. We agree with these literature findings that dyslipidemia is a well-known as a risk factor of ischemic stroke, and especially hyperlipidemia is higher in stroke patients than healthy controls.^[15-17]^ Coban et al. suggested that TG and TG/HDL-C were higher in young ischemic stroke patients than older stroke patients and controls regarding to recurrent ischemic stroke.^[17]^ These all articles are risk factor studies and they are important to prevent from secondary ischemic stroke, but the protection studies and acute-term mortality studies seem to be different from each other. The acute-phase stroke outcome studies are rare in the literature^[18]^ and mostly have different results from the classic atherosclerotic process.

The previous meta-analyses and reviews have generally concluded that there is no consistent positive association between cholesterol and stroke mortality.^[4,19,20]^ On the other hand, some studies demonstrated a correlation between lower TC levels and worse stroke outcome.^[18,21-25]^

The higher HDL-C level is another speculation. Sanossian et al.^[26]^ suggested that low-serum HDL was not associated with stroke severity. On the other hand, Chen et al. suggested that high acute-phase HDL-C had association with 30-day mortality in HS, and with stroke severity in ischemic stroke.^[21]^ In our study, HDL-C is insignificantly higher in mortality group.

Chen et al.^[21]^ suggested that low TC/HDL-C ratio had association with both 30-day and 1-year mortality in AIS. In our study, TC/HDL-C is significantly lower in the mortality group than surviving group, while TC is not significant (Table 1). However, the lower TC/HDL-C and TC levels are not independent predictors for mortality (Tables 3 and 4).

**Table 4.** Cholesterol and TC/HDL-C levels and regression analyses in survived and mortality groups in literature

	**Survived**	**Mortality**	**Cut-off point**	**HR (95%CI)**	**P**
Chen et al.^[21]^					
Cholesterol	168.21 (156–193)	160.48 (150–181)	<155.5/>192.2	0.35 (0.18–0.68)	0.002*
TC/HDL-C	3.54 (3.15–4.31)	3.24 (2.79–3.76)	>4.09	0.23 (0.10–0.50)	<0.001*
Cheng et al.^[18]^					
Cholesterol	179 (154–206)	160 (133–198)	<163.5	2.05 (1.47–2.86)	<0.001**
TC/HDL-C	4.3 (3.4–5.2)	3.8 (3.2–5.0)	<4.06	1.58 (1.13–2.23)	0.009**
Our study					
Cholesterol	177.5 (145–203)	168.5 (144–195)	≤170.5	1.484 (0.826–2.665)	0.186
TC/HDL-C	4.82 (3.9–6.0)	4.25 (3.6–5.2)	≤4.32	1.202 (0.619–2.333)	0.586

*Cholesterol quartile 1 (reference for regression, <155.5 mg/dL) to quartile 4 (/>192.2 mg/dL); *,** TC and TC/HDL-C rate are independent variables for mortality group. Cholesterol (mg/dL). high TC/HDL: Total cholesterol/high density lipoprotein; CI: Confidence interval.

On the other hand, TG and TG/HDL-C are significantly lower in the mortality group than surviving group (Table 1). TG is an independent predictor (<100.2 mg/dL) for mortality as well as TG/HDL-C (<2.56) (Table 3). Deng et al.^[2]^ observed that a lower TG/HDL-C value was significantly associated with 3-month mortality in the training and test cohort patients. The association of low TG with mortality after stroke may be consistent with a nutritional deficiency element. Serum TG is a better indicator of poor nutritional status.^[27]^ Weir et al.^[27]^ have shown that low fasting serum TG but not low cholesterol is a strong and independent predictor of mortality following stroke. The results of our study are similar to the study of Weir et al.^[27]^

Our retrospective study showing that the lower TG level is independent predictor in short-term mortality has insufficient to demonstrate the relationship between the lower TG-cholesterol levels and mortality in the etiological basis. However, we can suggest some following theories that show the relationship between lower TG and cholesterol and mortality, for the future prospective studies in the light of the literature.

Acute ischemic stroke is a hyper-catabolic severe trauma including inflammation, metabolic stressing, vulnerability, sarcopenia, and high-energy requirement, so that patients need the help of cholesterol and TG in the repairing process.^[18]^ Life-threatening stress in acute-phase stroke might cause increased TG and cholesterol consumption. This condition may be important for acute phase stroke outcome and may explain the speculative results in the literature.^[18]^

Cholesterol is a precursor of stress hormones, such as cortisol. Cholesterol is also an essential element of cell membranes, which cause intracellular transport, and cell signaling.[28] On the other hand, TGs store excess calories and provide energy in necessary conditions such as metabolic stress. It consists of one glycerol and three fatty-acids and is hydrolyzed by lipoprotein lipase enzyme (9.1 kcal/g).^[29]^

Malnutrition is encountered in neurologic diseases about 55–65%, which cause immune-suppression, increased infection risk, cardiac and respiratory failure, prolonged inpatient duration, and morbidity and mortality.^[27,30]^ Cholesterol and TG are important in this pathway.

Acute ischemic stroke is one of the metabolic stress conditions. It is needed to estimate total energy expenditure. The estimation of resting energy expenditure (REE) or basal energy expenditure is the first step. So many studies have compared the predictive accuracy of equations (Harris-Benedict, Cunningham, Wang, WHO/NAO/FAO, and Schofield-HW equations) for REE.^[31-33]^ These results indicated that the Harris-Benedict equation showed the best fit to actual REE values,^[31-33]^ especially in acute ischemic stroke.^[32]^ However, all the formulae were not accurate enough to be employed at the individual level (large SD of bias).^[33-36]^

The Harris and Benedict formula and the others should be re-evaluated to prevent the large sd.^[37]^ Gender, age, weight, and height are used in the estimation of REE in Harris-Benedict formula.^[31]^ For example, REE is 1398 kcal for two patients with same properties (male, 60 kg, 170 cm, 50 years old, Harris benedict formula=66.5 + (13.75x weight [kg]) + 5.003x height (cm)-(6.7775x age [year]36), but one patient has low serum TG level (80 mg/dL) and the other has high serum TG level (400 mg/dL). Is it similar condition regarding to glucose storage? Our patients had taken about 25–30 kcal/kg in the following period in the study. We detected that the mortality rate was 14.78 % in TG <100.0 mg/dL tertile, while 2.63% in the TG>200 mg/dL tertile in our study (Table 2). The question comes into mind: Why do not we use basal TG level in the REE estimation, while most formulations include in their basal serum levels such as glucose, sodium, and calcium, for example, TG is glucose stores and it is needed in the severe metabolic stress. TG <100.2 mg/dL is founded as an independent risk factor in the acute-term stroke mortality (Table 3) in the study.

### Limitations

This is not a malnutrition study, and it has not got any information about the pre- and post-stroke feeding status of the patients. Weight loss, body-mass index, prealbumin, retinol banding globulin, indirect calorimetry, and anthropometric measurements were missing to evaluate protein-energy malnutrition in our retrospective study. On the other hand, since the routine serum lipid panel is not frequently examined in our clinics, our patients do not have a control lipid panel for malnutrition in the acute period. A probable relationship between the low lipid levels, malnutrition and mortality may be hypothesis for future prospective studies. Antihyperlipidemic treatment effect could not be examined because of the low percentage of statin use (3.6%, Table 1).

## Conclusion

Dyslipidemia is a well-known risk for ischemic stroke. However, AS is a metabolic stress and malnutrition is one of the major problems. The decreased TG and TG/HDL rather than TC and TC/HDL seem to be an independent predictor for poor outcome in acute ischemic stroke. It is the hypothesis that basal TG levels should be taken into account for basal energy requirement in feeding formulas such as Harris-Benedict and the others. Prospective future studies are needed for not only AS but also the other metabolic stress factors.

### Disclosures

**Ethics Committee Approval:** Necmettin Erbakan University, Meram Medical Faculty, 185/2011.

**Peer-review:** Externally peer-reviewed.

**Conflict of Interest:** None declared.

**Authorship Contributions:** Concept – O.S.T., F.G., Desing – O.S.T., A.K., Supervision – O.S.T., F.G., A.K., Materials – O.S.T., A.B., Data collection&/or processing – E.E., H.B., A.S., Analysis and/or interpretetion – O.S.T., M.S.I., Literature search – O.S.T., A.B., Writing – O.S.T., F.G., Critical review – A.K.
